# Novel Long Noncoding RNA 005620 Induces Epirubicin Resistance in Triple-Negative Breast Cancer by Regulating ITGB1 Expression

**DOI:** 10.3389/fonc.2021.592215

**Published:** 2021-03-04

**Authors:** Fengliang Wang, Sujin Yang, Mingming Lv, Fei Chen, Hong Yin, Sheng Gao, Jinhai Tang, Jing Yu

**Affiliations:** ^1^ Department of Breast Surgery, The Affiliated Obstetrics and Gynaecology Hospital of Nanjing Medical University, Nanjing Maternity and Child Health Care Hospital, Nanjing, China; ^2^ Department of General Surgery, The First Affiliated Hospital of Nanjing Medical University, Nanjing, China; ^3^ Division of Geriatric Endocrinology, Department of Geriatrics, The First Affiliated Hospital of Nanjing Medical University, Nanjing, China

**Keywords:** triple-negative breast cancer, epirubicin resistance, long noncoding RNA, ITGB1, MDA-MB-231

## Abstract

Triple-negative breast cancer (TNBC) is often treated with anthracyclines (e.g., epirubicin or doxorubicin), but very little is known about anthracycline resistance, especially epirubicin resistance in TNBC. To identify novel long noncoding RNAs (lncRNAs) involved in epirubicin resistance in TNBC, we established a new TNBC MDA-MB-231 cell line that was resistant to epirubicin (Epi-R). A total of 12 differentially expressed lncRNAs were identified using RNA sequencing analysis of Epi-R cells. Among these lncRNAs, we found a novel intronic lncRNA, lnc005620, was highly expressed in Epi-R cells and human TNBC tissues. Further gain- and loss-of-function studies demonstrated that lnc005620 played an oncogenic role and partially abrogated the effects of epirubicin on TNBC cells. Using iTRAQ proteomics analysis, we found that three members of the integrin family, integrin β4, integrin β1 and integrin α6, were all upregulated in Epi-R MDA-MB-231 cells. Integrin β1, encoded by the ITGB1 gene, was validated to be a downstream target of lnc005620 in Epi-R MDA-MB-231 cells. Our study demonstrates that novel lnc005620 promotes TNBC progression and chemoresistance to epirubicin *via* integrin β1 both *in vitro* and *in vivo* and provides a promising therapeutic target for TNBC patients in terms of enhancing the benefits of epirubicin treatment.

## Introduction

Breast cancer is the most commonly diagnosed cancer and the second leading cause of cancer death in women worldwide (1). Triple-negative breast cancer (TNBC), which constitutes approximately 12–17% of breast cancer cases, is a heterogeneous subtype characterized by the absence of estrogen receptor (ER), progesterone receptor (PR), and human epidermal growth factor receptor 2 (HER2) (2). Compared to other subtypes, TNBC is usually more aggressive and associated with the development of resistance to conventional chemotherapeutics (2, 3). Patients with TNBC experience worse prognosis and shorter overall survival owing to higher rates of recurrence, early metastasis and limited therapeutic options (4).

Anthracyclines (ANTs), including doxorubicin, epirubicin and idarubicin, are the main therapeutic drugs for TNBC (5). The currently known antitumor mechanisms of ANTs are the inhibition of DNA and RNA synthesis, thus preventing the replication of rapidly growing cancer cells (6). ANTs also have the capacity to improve the host immune system to boost the efficacy of chemotherapy (7, 8). Among ANTs, epirubicin is the most effective against TNBC, but 30–40% of patients still respond poorly, and acquired resistance has been reported (5, 9). Although preclinical models suggest that drug transport proteins (10, 11), antioxidant defenses (12–14), apoptotic signaling (15–17), and topoisomerase modulation (18, 19) may mediate ANT resistance, much less is known about ANT resistance, especially epirubicin resistance of TNBC, than about other drugs. Hence, it is urgent to reveal the potential factors involved in regulating epirubicin resistance in TNBC and find useful therapeutic targets for patients.

Due to the development of high-throughput technologies, such as microarrays and next-generation sequencing (NGS), an increasing number of novel transcripts have been detected, and the vast majority of these transcripts do not seem to be derived from annotated protein-coding genes (20). Among the various types of non-protein-coding transcripts, long noncoding RNAs (lncRNAs), which are more than 200 nucleotides in length, have attracted increasing attention (21, 22). lncRNAs have been proposed to carry out diverse functions, including transcriptional regulation, organization of nuclear domains, and regulation of proteins or RNA molecules (23). Thus, it is not surprising that lncRNAs have been implicated in diseases. An increasing number of studies have revealed the ability of lncRNAs to modulate a variety of oncogenic processes, including tumor formation and metastatic progression (24, 25). The involvement of lncRNAs in the development of chemoresistance in breast cancers has also been reported (26, 27). Therefore, it is meaningful to investigate the roles of lncRNAs in regulating the tumorigenesis and drug resistance of TNBC, which can help with the identification of novel therapeutic targets.

In our study, to identify novel lncRNAs involved in epirubicin resistance in TNBC, we established a new TNBC MDA-MB-231 cell line that is resistant to epirubicin. A novel intronic lncRNA, lnc005620, whose host gene was DnajB6, a negative regulator of breast cancer, was discovered using RNA sequencing analysis. lnc005620 was highly expressed in Epi-R cells and human TNBC tissues. Further gain- and loss-of-function studies demonstrated that lnc005620 played an oncogenic role and partially abrogated the effects of epirubicin on TNBC cells. FISH assays showed that lnc005620 was mainly distributed in the cytoplasm of MDA-MB-231 cells. The cell surface receptor integrin β1 was found to be the downstream target of lnc005620 *via* iTRAQ proteomic analysis. The role of lnc005620 in facilitating tumorigenesis and epirubicin resistance was also validated *in vivo*.

## Materials and Methods

### Patients Samples and Study Approval

Primary cancer tissue and adjacent non‐cancerous tissue samples were all from the patients of Department of Breast Surgery, the Affiliated Obstetrics and Gynaecology Hospital of Nanjing Medical University. Immediately after excision, samples were transported to the laboratory. All the patients were pathologically confirmed. Human study was approved by the Ethics Committee of the Affiliated Obstetrics and Gynaecology Hospital of Nanjing Medical University. Written permission was obtained in all cases from the donor’s family to use breast cancer tissues for experimental research.

Animal welfare and experimental procedures were carried out strictly in accordance with National Research Council guidelines for the care and use of laboratory animals (1996) and were reviewed and approved by the Laboratory Animal Welfare Ethics Committee of Nanjing Medical University.

### Cell Culture and Reagents

The human triple-negative breast cancer cell line MDA-MB-231 was purchased from Chinese Type Culture Collection, Chinese Academy of Sciences (Shanghai, China). Short tandem repeat (STR) typing profiles of the cell line were analyzed to identify the cell origin and detect intraspecies cross-contamination of human origin. MDA-MB-231 cells were cultured in L15 medium (Gibco, NY, USA) supplemented with 100 U/ml penicillin/streptomycin (Thermo Fisher, MA, USA) and heat inactivated 10% fetal bovine serum (FBS, Gibco) at 37°C in a humidified incubator with 5% CO_2_.

Epirubicin (Famaxin^®^) was obtained from Pfizer (NY, USA) and dissolved in PBS. Epirubicin-resistant MDA-MB-231 cells were generated by exposing native cells to increasing concentrations of epirubicin (0, 6.25, 31.25 and 62.5 ng/ml). Cells were treated for 8 h every time, and after eight rounds of repeated intermittent induction as described before (“clinically relevant model”—pulsed treatment to mimic the cycles of chemotherapy) (28), MDA-MB-231 cells resistant to 31.25 ng/ml epirubicin were obtained. Resistance was defined when the half-maximal inhibitory concentration (IC_50_) value superseded the IC_50_ value of the corresponding native cell line and resistant cells could not tolerate a further increase in drug concentration. Cell viability was determined using the MTT Cell Proliferation and Cytotoxicity Assay Kit (Beyotime, Shanghai, China). The IC_50_ value was calculated using Prism 8 software (GraphPad Software, CA, USA).

### RNA Isolation, Library Preparation, Sequencing and Data Analysis

Total RNA from epirubicin-resistant and native MDA-MB-231 cells was extracted using TRIzol™ Reagent (Thermo Fisher) according to the manufacturer’s instructions. RNA purity was tested using a NanoPhotometer^®^ spectrophotometer (IMPLEN, CA, USA). RNA concentration was measured using a Qubit^®^ RNA Assay Kit in Qubit^®^ 2.0 Fluorometer (Life Technologies, CA, USA). RNA integrity was assessed using the RNA Nano 6000 Assay Kit and the Bioanalyzer 2100 system (Agilent Technologies, CA, USA). See **Supplementary Methods** in the online Supporting Information for details.

### Protein Extraction, iTRAQ Proteome and Data Analysis

Samples for RNA sequencing were also used to perform proteome analysis. Proteins were extracted as previously described (29). See **Supplementary Methods** in the online Supporting Information for details.

### Fluorescence *In Situ* Hybridization (FISH)

The FISH probe specific to lnc005620 was designed and synthesized (Ribo, Guangzhou, China) according to the manufacturer’s instructions. See **Supplementary Methods** in the online Supporting Information for details.

### Cell Transfection

The full-length of lnc005620 (**Supplementary Table 1**) was amplified and cloned into the vector pcDNA3.1 by GeneCreat (Wuhan, China). Small interfering RNA (siRNA) sequences targeting lnc005620 or ITGB1 (**Supplementary Table 2**) were also designed and synthesized by GeneCreat. Cells were cultured in medium until 80% confluence and transfected. Transfection was carried out using Lipofectamine 2000 (Invitrogen, CA, USA) following the manufacturer’s instructions. Transfection efficiency was detected by RT-qPCR 24 h later.

### Cell Viability Assays

The altered cell viability was assayed using the Cell Counting Kit-8 (CK04, Dojindo, Rockville, MD, USA). In brief, cells were seeded into a 96‐well plate and treated with the CCK8 reagent and further cultured for 0.5 h. The optical density at 450 nm was measured with a Multiscan Spectrum (MB-580, Huisong, Shenzhen, China).

EdU assay was used to measure the rate of cell proliferation. According to the manufacturer’s instructions (BeyoClick™ EdU Cell Proliferation Kit with Alexa Fluor 594, C0078S, Beyotime), cells were cultured in a 24‐well plate and treated with 20 μM EdU. The nuclei were stained with Hoechst dye 33,342. Images of five randomly selected areas of each group were taken with an UltraVIEW^®^ VoX system (PerkinElmer, IL, USA).

### Flow Cytometry

Flow cytometric analysis was performed to evaluate cell apoptosis. In brief, cells were collected followed by staining with Annexin V-FITC/PI according to the manufacturer’s instructions (Annexin V-FITC/PI Apoptosis Detection Kit, C1062, Beyotime). Data were collected using a CytoFLEX flow cytometer and CytExpert software (Beckman Coulter, CA, USA).

### Cell Migration and Invasion Assays

A wound healing assay was used to evaluate cell migration. Wounds were scratched on the cell monolayer using 20 μl pipette tips. Non-adherent cells were removed by washing with PBS, and then the cells were cultured for 48 h and imaged under a microscope (Olympus cx41, Tokyo, Japan). Adobe Photoshop CS6 (Adobe Systems, CA, USA) was used for the quantitative analysis.

Cell invasion was detected using a transwell invasion assay. In brief, 100 μl Matrigel (BD, NY, USA) was first added to the bottom of the transwell chamber (24-well insert, TCS003024, Jet Biofil, Guangzhou, China), and then 1 × 10^5^ cells in FBS-free medium were placed on the membrane in the chamber. Migrated cells on the permeable membrane were fixed using 4% formaldehyde, stained with crystal violet, and imaged under a microscope (Olympus cx41). ImageJ V1.8.0 (NIH, MD, USA) was used for quantitative analysis.

### Immunohistochemistry and Immunofluorescence

Human breast cancer tissues were fixed in 10% formalin, processed, and paraffin embedded. Multiple sections (5 mm) were prepared. See **Supplementary Methods** in the online Supporting Information for details.

### Reverse Transcription-Quantitative Polymerase Chain Reaction (RT-qPCR)

Total RNA was extracted using TRIzol™ Reagent (Thermo Fisher) according to the manufacturer’s instructions. Two micrograms of RNA were reverse transcribed with SuperScript III^®^ (Thermo Fisher). The obtained cDNA was quantified by using SYBR Green Real-time PCR Master Mix (Roche, Basel, Switzerland). See **Supplementary Methods** in the online Supporting Information for details.

### Protein Extraction and Western Blotting

Total protein was extracted, and the concentration was determined by the Bradford method. See **Supplementary Methods** in the online Supporting Information for details.

### *In Vivo* Tumorigenesis Assay

The full-length lnc005620 was amplified and cloned into a lentivirus vector for retrovirus production in MDA-MB-231 cells (Lv–lnc005620). Male BALB/c nude mice (5 weeks of age) were purchased from Beijing Vitalriver Laboratory Animal Co., Ltd (Beijing, China). MDA-MB-231 cells (5 × 10^6^) transfected with Lv–lnc005620 or Lv–NC (empty vector for negative control) were suspended in 200 μl PBS and then injected subcutaneously in the mouse flanks. When tumors were palpable, the mice were randomized into epirubicin (Pharmorubicin^®^, Pfizer) treatment groups or control groups. Epirubicin was dissolved in PBS and injected subcutaneously at the tumor sites (5 mg/kg) weekly. Treatment lasted for 2 weeks until the xenograft tumor was removed and the mass was calculated.

### Statistics

Data are presented as the mean ± SD. Significant differences between two groups were analyzed using two-tailed, unpaired *t*-tests. One-way ANOVA was used for comparisons between multiple groups. GraphPad Prism software (version 8.0.0; GraphPad Software Inc., CA, USA) was used for statistical analysis. *P* < 0.05 was considered statistically significant.

## Results

### Characterization of Epirubicin-Resistant MDA-MB-231 Cells

We initiated our study by identifying the MDA-MB-231 cell line through STR typing profile analysis (**Supplementary Table 3** and **Supplementary Figure 1**) and generating an epirubicin-resistant MDA-MB-231 cell line. Native and resistant cells were exposed to epirubicin concentrations ranging from 6.25 to 3,125 ng/ml. Cell viability was determined 48 h later by MTT assay (**Supplementary Figure 2**). The IC_50_ values of MDA-MB-231 native and resistant cells were 0.26 ng/ml and 1.4 ng/ml, respectively. The resistance index was calculated by the resistant IC_50_/native IC_50,_ and the value was 5.38.

**Figure 1:**
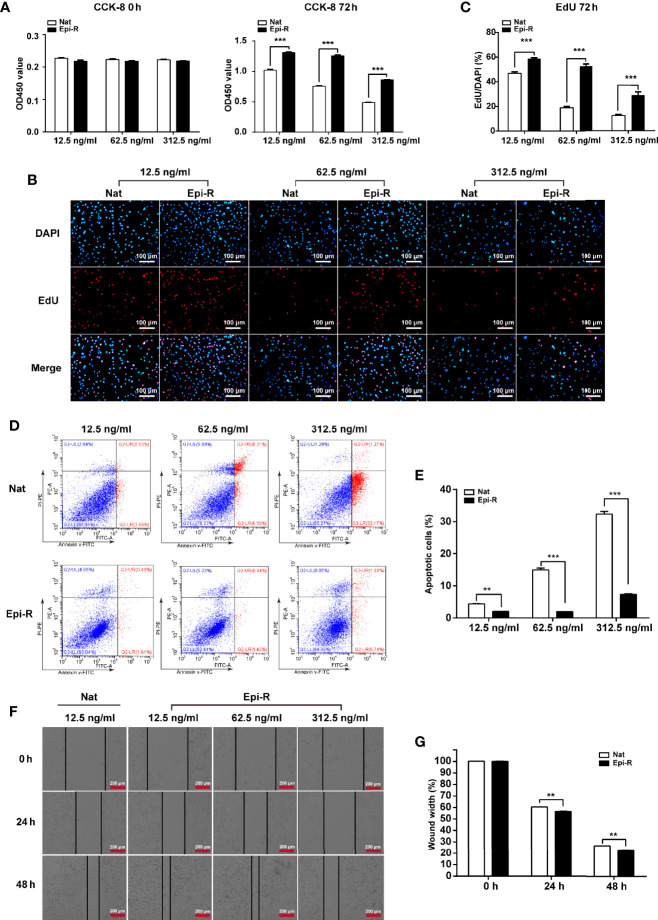
Characterization of epirubicin-resistant MDA-MB-231 cells. Epirubicin-resistant (Epi-R) and native (Nat) MDA-MB-231 cells were exposed to different concentrations of epirubicin (12.5 ng/ml, 62.5 ng/ml and 312.5 ng/ml). **(A)** Cell proliferation analysis by CCK-8 assay. **(B)** Cell proliferation analysis by EdU assay. DAPI for nuclei. Scale bars: 100 μm. **(C)** Quantitative analysis of EdU assay. **(D)** Flow cytometry analysis of cell apoptosis. **(E)** Quantitative analysis of apoptotic cell percentages. **(F)** Cell migration analysis by wound healing assay. Scale bars: 200 μm. **(G)** Quantitative analysis of cell migration. n = 3. Data are represented as the mean ± SD, **P < 0.01, ***P < 0.001.

To determine the characteristics of epirubicin-resistant MDA-MB-231 cells, CCK-8 and EdU assays were performed after 72 h of exposure to epirubicin at concentrations of 12.5 ng/ml, 62.5 ng/ml and 312.5 ng/ml. We demonstrated that epirubicin-resistant MDA-MB-231 cells showed elevated cell proliferation compared to the native controls (**Figures 1A–C**). Further, apoptotic cells were identified by flow cytometry after 48 h of exposure to epirubicin. The percentages of apoptotic cells were consistently lower in epirubicin-resistant MDA-MB-231 cells than the native controls (**Figures 1D, E**). Subsequently, we determined the effect of epirubicin on cell migration. The wound healing assay indicated that the native MDA-MB-231 cells died more rapidly and could not completely cover the scratches after the cells were treated with epirubicin at concentrations of 62.5 ng/ml and 312.5 ng/ml. The migratory ability of epirubicin-resistant MDA-MB-231 cells was higher than that of the native controls at a concentration of 12.5 ng/ml after treatment of epirubicin for 24 h or 48 h (**Figures 1F, G**). According to the above results, we verified the characteristics of epirubicin-resistant MDA-MB-231 cells.

### Identification of Novel lncRNAs in Epirubicin-Resistant MDA-MB-231 Cells

**Figure 2:**
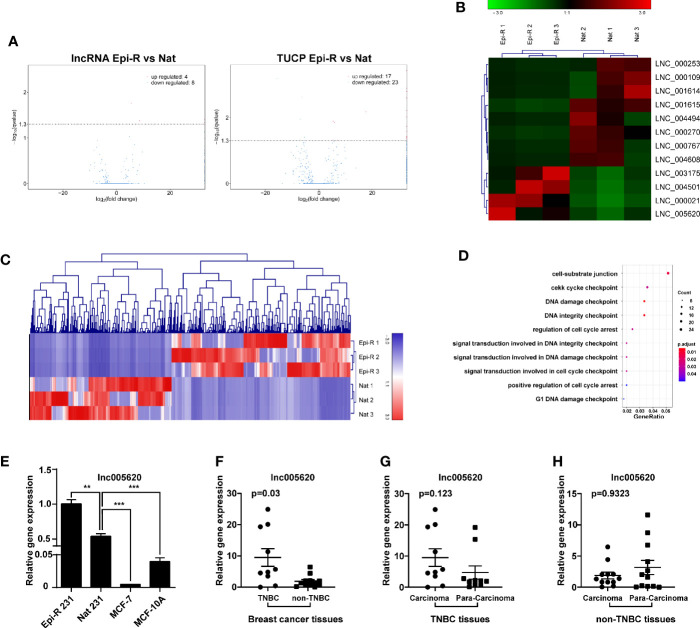
Identification of novel lncRNAs in epirubicin-resistant MDA-MB-231 cells. Epirubicin-resistant (Epi-R) and native (Nat) MDA-MB-231 cells were used for RNA sequencing analysis. **(A)** Volcano plots of differentially expressed lncRNAs and transcripts of uncertain coding potential (TUCPs) between Epi-R and Nat cells. **(B)** Hierarchical cluster analysis diagram of 12 novel differentially expressed lncRNAs. n = 3. **(C)** Hierarchical clustering analysis diagram of 521 coexpressed genes of lnc005620. **(D)** GO functional enrichment analysis of 521 genes coexpressed with lnc005620. n = 3. **(E)** RT-qPCR validation of lnc005620 expression in Epi-R and Nat MDA-MB-231 cells, MCF-7 cells and MCF-10A cells. n = 3. **(F–H)** RT-qPCR analysis of lnc005620 expression in breast cancer tissues and paired adjacent non‐tumor tissues from TNBC (n =10) and non-TNBC (n = 12) patients. Data are represented as the mean ± SD, **P < 0.01, ***P < 0.001.

To identify lncRNAs associated with epirubicin resistance in TNBC chemotherapy, we performed whole transcriptome sequencing (RNA-Seq) using RNA from epirubicin-resistant MDA-MB-231 cells and their native controls (GEO accession number: GSE152003). After strict analysis and screening of the sequencing results, we obtained a total of 52 differentially expressed transcripts (**Figure 2A**). Coding potential analysis software was used to screen transcripts of uncertain coding potential (TUCP), and we found 40 TUCPs, of which 17 were upregulated and 23 were downregulated in epirubicin-resistant cells (**Figure 2A**). A total of 12 differentially expressed lncRNAs were obtained after excluding these TUCPs, of which four lncRNAs were upregulated and eight were downregulated in epirubicin-resistant cells compared with the native controls (**Figure 2B** and **Table 1**).

**Table 1:** Differentially expressed lncRNAs of epirubicin-resistant and native MDA-MB-231 cells identified by RNA-Seq.

Novel_lncRNA_ID	Gene_Type	Chromosome	Start	End	Strand	Length(bp)	fold change	*q* value	analyze
LNC_000021	lincRNA	GL000220.1	112106	157660	+	1690	5.50	0.017	up
LNC_005620	intronic_lncRNA	chr7	1.57E+08	1.57E+08	−	2511	8.75	0.042	up
LNC_004501	intronic_lncRNA	chr4	73404373	73421223	−	1940	Inf	0.046	up
LNC_003175	lincRNA	chr2	1.13E+08	1.13E+08	+	4477	Inf	0.039	up
LNC_004494	antisense	chr4	55878135	55948105	−	4044	#NAME?	0.049	down
LNC_004608	intronic_lncRNA	chr5	6599280	6633281	+	3238	#NAME?	0.018	down
LNC_000109	lincRNA	KI270733.1	148201	173452	−	415	#NAME?	0.005	down
LNC_000253	intronic_lncRNA	chr1	77714596	77759962	+	2759	#NAME?	0.003	down
LNC_000270	intronic_lncRNA	chr1	86352171	86396310	+	2022	#NAME?	0.010	down
LNC_000767	intronic_lncRNA	chr1	1.84E+08	1.84E+08	−	3815	#NAME?	0.045	down
LNC_001614	intronic_lncRNA	chr12	1.12E+08	1.12E+08	+	918	#NAME?	0.007	down
LNC_001615	intronic_lncRNA	chr12	1.17E+08	1.17E+08	+	1394	#NAME?	0.019	down

lncRNAs can have a regulatory effect on adjacent protein-coding genes, so first we performed a colocalization gene analysis of the 12 differentially expressed lncRNAs, and the colocalization threshold was set to 100 kb upstream and downstream of lncRNAs. Second, we conducted a coexpression analysis by analyzing the correlation coefficient between lncRNA and mRNA expression to predict the target genes of lncRNAs. Finally, GO analysis was used to clarify the function of these mRNAs and to predict the potential roles of the lncRNAs. Among the 12 novel lncRNAs was the intronic lncRNA lnc005620, which was located on chromosome 7 and had a total length of 2,511 bp. Based on the colocalization analysis, we found that the host gene of lnc005620 was DnaJB6, a member of the DnaJ/Hsp40 family. DnaJB6 is a negative regulator of breast tumor formation and metastasis (30). Further coexpression analysis revealed that 521 genes were coexpressed with lnc005620 (**Supplementary Table 4**). Heat-map clustering analysis of these coexpressed genes is shown in **Figure 2C**. GO functional enrichment analysis of these genes showed that the top ten functions were mainly focused on the regulation of cell cycle arrest and DNA damage (**Figure 2D**). Based on this information, our further research was focused on lnc005620.

To identify the noncoding characteristics of lnc005620, the National Genomics Data Center (NGDC, https://bigd.big.ac.cn/lgc/calculator) and Coding Potential Calculator (http://cpc.gao-lab.org/programs/run_cpc.jsp) databases were used to calculate the coding potential score of lnc005620. The values were −0.265 and −1.00201, respectively, which were both less than zero. A coding potential score of a transcript that is greater than zero indicates a protein-coding RNA, while if it smaller than zero, it indicates a ncRNA. We also investigated the open reading frames (ORFs) of lnc005620 that might encode peptides by BLAST search (https://www.ncbi.nlm.nih.gov/orffinder) and found that none were similar to the amino acid sequence of the existing protein.

To confirm that the expression of lnc005620 was altered in epirubicin-resistant cells, we detected the expression level of lnc005620 in epirubicin-resistant MDA-MB-231 cells. A significantly elevated expression of lnc005620 was detected in epirubicin-resistant MDA-MB-231 cells compared to their native controls (**Figure 2E**). We also measured the expression of lnc005620 in the non-triple negative breast cancer (non-TNBC) cell line MCF-7 and the human normal breast epithelial cell line MCF-10A and found lower levels of lnc005620 in both cell lines (**Figure 2E**). Then, we detected the expression of lnc005620 in 22 breast cancer tissues and paired adjacent non-tumor tissues, among which 10 were TNBC and 12 were non-TNBC (**Supplementary Table 5**). The results showed that compared with that in non-TNBC tissues, the expression of lnc005620 in TNBC tissues was significantly increased (**Figure 2F**). There was no difference in the expression of lnc005620 between the cancer tissues and paired adjacent tissues in both TNBC and non-TNBC (**Figures 2G, H**). Taken together, our data indicated that lnc005620 might have potential roles in regulating epirubicin resistance in TNBC.

### lnc005620 Promotes Proliferation, Invasion and Epirubicin Resistance in MDA-MB-231 Cells

**Figure 3:**
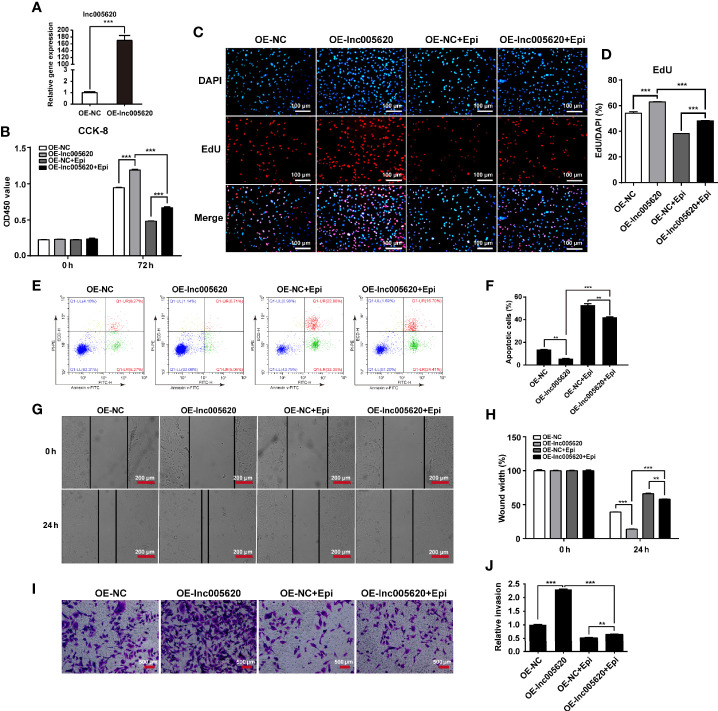
lnc005620 promotes proliferation, invasion and epirubicin resistance in MDA-MB-231 cells. lnc005620 was overexpressed in native MDA-MB-231 cells treated with or without 12.5 ng/ml epirubicin. **(A)** RT-qPCR validation of lnc005620 expression. **(B)** Cell proliferation analysis by CCK-8 assay. **(C)** Cell proliferation analysis by EdU assay. DAPI for nuclei. Scale bars: 100 μm. **(D)** Quantitative analysis of EdU assay. **(E)** Flow cytometry analysis of cell apoptosis. **(F)** Quantitative analysis of apoptotic cell percentages. **(G)** Cell migration analysis by wound healing assay. Scale bars: 200 μm. **(H)** Quantitative analysis of cell migration. **(I)** Cell invasion analysis by Transwell assay. Scale bars: 500 μm. **(J)** Quantitative analysis of cell invasion. OE-NC, negative control; OE-lnc005620, overexpression of lnc005620; OE-NC+Epi, negative control and exposure to epirubicin; OE-lnc005620+Epi, overexpression of lnc005620 and exposure to epirubicin. n = 3. Data are represented as the mean ± SD, **P < 0.01, ***P < 0.001.

We then investigated the functional role of lnc005620 in TNBC progression and epirubicin resistance. According to the high expression of lnc005620 in epirubicin-resistant MDA-MB-231 cells, we constructed a lnc005620 overexpression model using native MDA-MB-231 cells to study the role of lnc005620 in the proliferation, apoptosis and invasion of MDA-MB-231 cells (**Figure 3A**). According to the results from CCK-8 and EdU assays, we found that MDA-MB-231 cells overexpressing lnc005620 showed elevated cell proliferation compared to the control group (**Figures 3B–D** ). After treatment with 12.5 ng/ml epirubicin for 72 h, cell proliferation decreased but was still higher in cells overexpressing lnc005620 (**Figures 3B–D** ). Using flow cytometry, we found that MDA-MB-231 cells overexpressing lnc005620 showed significantly lower percentages of apoptotic cells (**Figures 3E, F**). The percentage of apoptotic cells increased but was still lower in cells overexpressing lnc005620 after 48 h exposure to 12.5 ng/ml epirubicin (**Figures 3E, F**). Furthermore, in the wound healing assay, the results indicated that overexpression of lnc005620 promoted cell migration (**Figures 3G, H**). As expected, migratory ability was inhibited after 24 h treatment of epirubicin but remained higher in cells overexpressing lnc005620 (**Figures 3G, H**). Next, the Transwell assay also showed that lnc005620 promoted the invasion of MDA-MB-231 cells regardless of whether the cells were treated with epirubicin for 48 h (**Figures 3I, J**). These results demonstrated that lnc005620 played an oncogenic role and partially abrogated the effects of epirubicin on MDA-MB-231 cells.

### Inhibition of lnc005620 Alleviates Proliferation, Invasion, and Epirubicin Resistance in MDA-MB-231 Cells

**Figure 4:**
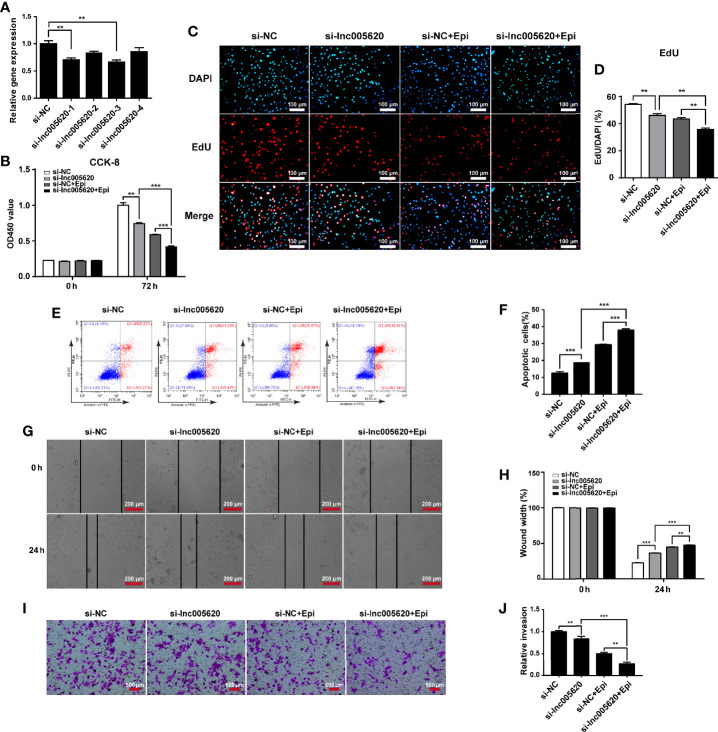
Inhibition of lnc005620 alleviates proliferation, invasion and epirubicin resistance in MDA-MB-231 cells. lnc005620 was knocked down in epirubicin-resistant MDA-MB-231 cells treated with or without 12.5 ng/ml epirubicin. **(A)** RT-qPCR validation of lnc005620 expression. **(B)** Cell proliferation analysis by CCK-8 assay. **(C)** Cell proliferation analysis by EdU assay. DAPI for nuclei. Scale bars: 100 μm. **(D)** Quantitative analysis of EdU assay. **(E)** Flow cytometry analysis of cell apoptosis. **(F)** Quantitative analysis of apoptotic cell percentages. **(G)** Cell migration analysis by wound healing assay. Scale bars: 200 μm. **(H)** Quantitative analysis of cell migration. **(I)** Cell invasion analysis by Transwell assay. Scale bars: 500 μm. **(J)** Quantitative analysis of cell invasion. si-NC, negative control; si-lnc005620, siRNA against lnc005620; si-NC+Epi, negative control and exposure to epirubicin; si-lnc005620+Epi, siRNA against lnc005620 and exposure to epirubicin. n = 3. Data are represented as the mean ± SD, **P < 0.01, ***P < 0.001.

To investigate whether inhibition of lnc005620 could alleviate epirubicin resistance in TNBC cells, we constructed a lnc005620 knockdown model by transfecting four siRNA sequences targeting lnc005620 into epirubicin-resistant MDA-MB-231 cells (**Figure 4A**). We found that knockdown of lnc005620 led to a decrease in proliferation of epirubicin-resistant MDA-MB-231 cells and this effect was more significant after treatment with 12.5 ng/ml epirubicin for 72 h (**Figures 4B–D**). An increase in the apoptotic cell percentage in epirubicin-resistant cells was also demonstrated (**Figures 4E, F**). After treatment with 12.5 ng/ml epirubicin for 48 h, a further increase in the proportion of apoptotic cells was observed in lnc005620 knockdown cells (**Figures 4E, F**). Wound healing assays indicated that knockdown of lnc005620 weakened the migratory ability of epirubicin-resistant cells and enhanced the inhibitory effect of epirubicin on cell migration (**Figures 4G, H**). Transwell assays were used to determine whether inhibition of lnc005620 influenced cell invasion in epirubicin-resistant cells. As expected, knockdown of lnc005620 suppressed cell invasion and enhanced the anti-invasion effect of epirubicin (**Figures 4I, J**). To this end, we concluded that inhibition of lnc005620 alleviated epirubicin resistance in MDA-MB-231 cells.

To validate the effect of lnc005620 on other TNBC cell lines, we used another ANT, doxorubicin (Dox) and performed the studies in MDA-MB-436 and MDA-MB-468 cells. Firstly, we measured the expression of lnc005620 in these two cell lines and found a lower level of lnc005620 in MDA-MB-436 and MDA-MB-468 cells than epirubicin-resistant and native MDA-MB-231 cells (**Supplementary Figure 3A**). Then CCK-8 assay was used to detected cell proliferation in these cells after 48 and 72 h of exposure to doxorubicin at concentrations of 12.5, 62.5, and 312.5 ng/ml (**Supplementary Figures 3B, C**). According to the results, we did further research by using 62.5 ng/ml doxorubicin for 72 h. Next, we constructed a lnc005620 overexpression model using native MDA-MB-436 and MDA-MB-436 cells. Both of the two kinds of cells overexpressing lnc005620 showed increased proliferation, migration and invasion ability, and simultaneously decreased cell apoptosis with or without doxorubicin treatment (**Supplementary Figures 4**, **5**). These results furtherly verified the oncogenic role of lnc005620 in other TNBC cells treated with another ANT.

### Identification of Key Proteins Associated With Epirubicin Resistance in MDA-MB-231 Cells

**Figure 5:**
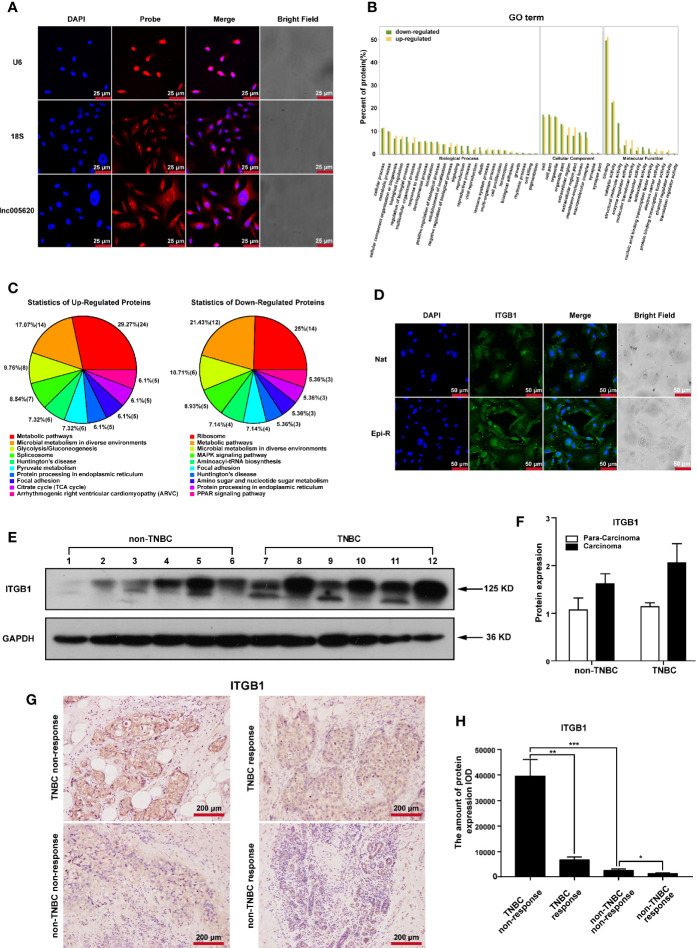
Identification of key proteins associated with epirubicin resistance in MDA-MB-231 cells. Epirubicin-resistant (Epi-R) and native (Nat) MDA-MB-231 cells were used for iTRAQ proteome analysis. **(A)** FISH analysis of the subcellular location of lnc005620 with a specific probe in native MDA-MB-231 cells. Small nuclear RNA U6 (U6) and 18S ribosomal RNA (18S) served as references for nuclear and cytoplasmic localization, respectively. Blue: DAPI for nuclei, red: probe for target genes. Scale bars: 25 μm. n = 3. **(B)** GO function analysis of 202 differentially expressed proteins between Epi-R and Nat cells. **(C)** KEGG analysis of 202 differentially expressed proteins between Epi-R and Nat cells. **(D)** Immunofluorescence staining of ITGB1 in Epi-R and Nat cells. Blue: DAPI for nuclei, green: ITGB1. Scale bars: 50 μm. n = 3. **(E)** Western blotting of ITGB1 in breast cancer tissues and paired adjacent non‐tumor tissues from TNBC and non-TNBC patients. Glyceraldehyde 3-phosphate dehydrogenase (GAPDH) was used as an internal reference. Lines 1, 3, 5, 7, 9, 11: para-carcinoma tissues; lines 2, 4, 6, 8, 10, 12: carcinoma tissues. **(F)** Quantitative analysis of ITGB1 protein expression. **(G)** Immunohistochemistry analysis of ITGB1 expression in breast cancer tissues from TNBC and non-TNBC patients responding or nonresponding to epirubicin treatment. Scale bars: 200 μm. n = 3/group. **(H)** Quantitative analysis of ITGB1 expression. Data are represented as the mean ± SD, *P < 0.05, **P < 0.01, ***P < 0.001.

To investigate the underlying functional proteins contributing to epirubicin resistance in TNBC and the possible mechanisms of action of lnc005620, proteome analysis was performed using the iTRAQ method. First, we detected the subcellular location of lnc005620 by using FISH assay with a specific probe. The results showed that lnc005620 was mainly distributed in the cytoplasm of MDA-MB-231 cells (**Figure 5A**). After iTRAQ proteomics analysis, a total of 202 differentially expressed proteins (DEPs) were obtained, of which 130 proteins were upregulated and 72 were downregulated in epirubicin-resistant cells compared with their native controls (**Supplementary Table 6**). GO function analysis (**Figure 5B**) demonstrated that DEPs were enriched in metabolic process and biological regulation within the biological process category. In the cellular component category, the organelle part and extracellular region were the dominant functions. In the molecular function category, binding and catalytic activity accounted for a major proportion. KEGG analysis of the DEPs (**Figure 5C**) was performed, and the results showed that 24 upregulated and 12 downregulated DEPs were enriched in metabolic pathways. The top ten pathways included focal adhesion and the MAPK signaling pathway.

Among these DEPs, we found that three members of the integrin family, integrin β4, integrin β1 and integrin α6, were all upregulated in epirubicin-resistant cells. Integrins comprise a large family of cell surface receptors that are composed of two subunits, α and β (31). The β1 integrin subunit encoded by the ITGB1 gene is one member of this large family and is a critical mediator of breast cancer initiation and progression (32, 33). Overexpression of ITGB1 has been associated with poor overall survival in TNBC patients (34). Here, we calculated the Pearson’s correlation coefficient of lnc005620 and ITGB1, and the value was 0.56 (*P* = 0.044). Considering that lnc005620 is mainly located in the cytoplasm and ITGB1 is a cell surface receptor, we focused on ITGB1 for further research on the mechanism of lnc005620.

To confirm the high expression of ITGB1 in epirubicin-resistant MDA-MB-231 cells, immunofluorescence was used, and we found that the expression of ITGB1 increased in epirubicin-resistant cells compared to the native cells (**Figure 5D**). Then, we detected the protein expression of ITGB1 in breast cancer tissues and paired adjacent non-tumor tissues from patients diagnosed with TNBC or non-TNBC. Western blotting analysis showed that an increased trend of ITGB1 expression was observed in cancer tissues from TNBC (**Figure 5E**), but quantitative analysis of the bands showed no significant difference (**Figure 5F**). Furthermore, 12 breast cancer tissues from patients who received epirubicin treatment were used for immunohistochemistry of ITGB1. Patients were divided into nonresponding (3 were TNBC and non-TNBC) and responding (3 were TNBC and non-TNBC) groups (**Supplementary Table 7**) according to the Response Evaluation Criteria In Solid Tumors (RECIST, version 1.1) (35). The results showed that ITGB1 was upregulated significantly in TNBC patients who did not respond to epirubicin treatment compared with those who showed a response to epirubicin therapy (**Figures 5G, H**). Silencing of ITGB1 suppresses TNBC cell migration and invasion (36). We also confirmed that knockdown of ITGB1 promoted apoptosis and inhibited the migration and invasion of epirubicin-resistant MDA-MB-231 cells (**Supplementary Figure 6**). In summary, ITGB1 plays an oncogenic role and might be associated with nonresponse to epirubicin treatment in TNBC patients.

### ITGB1 Is a Downstream Target of lnc005620 That Functions in the Epirubicin Resistance of MDA-MB-231 Cells

**Figure 6:**
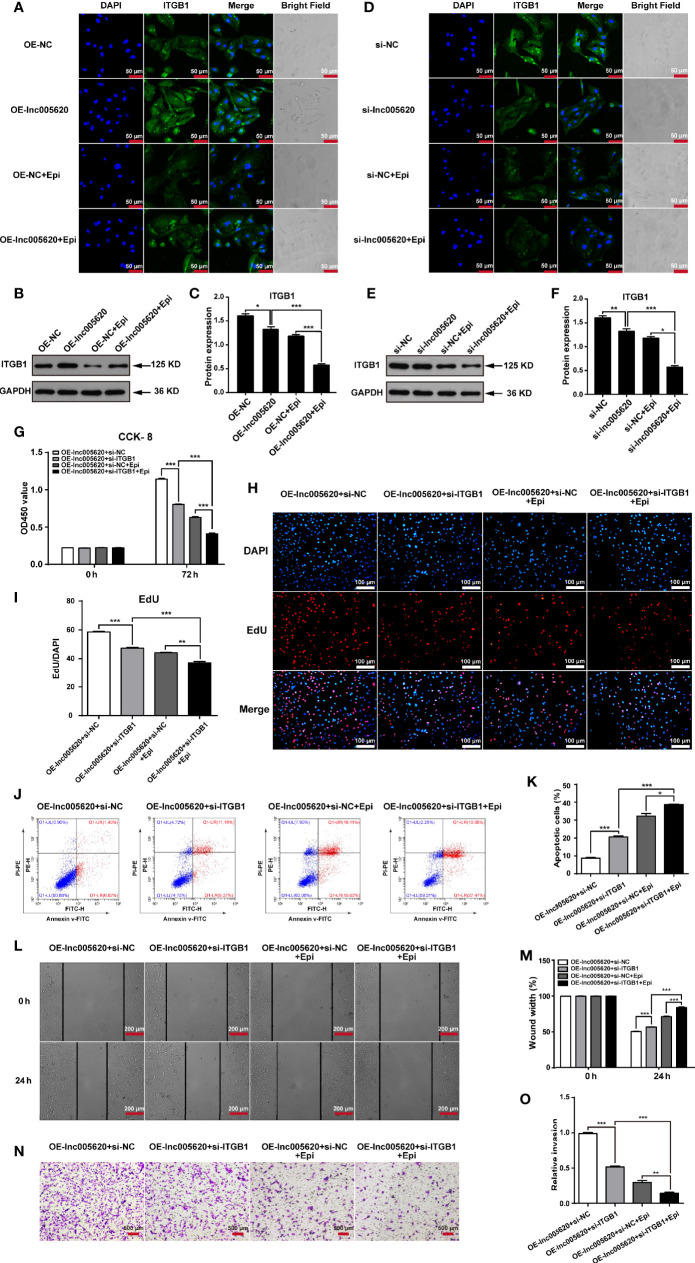
ITGB1 is a downstream target of lnc005620 that functions in the epirubicin resistance of MDA-MB-231 cells. lnc005620 was overexpressed in native MDA-MB-231 cells or knocked down in epirubicin-resistant MDA-MB-231 cells, and treated with or without 12.5 ng/ml epirubicin for 48 h **(A–F)**. **(A, D)** Immunofluorescence staining of ITGB1. Blue: DAPI for nuclei, green: ITGB1. Scale bars: 50 μm. **(B, E)** Western blotting of ITGB1. **(C, F)**. Quantitative analysis of ITGB1 protein expression. lnc005620 was overexpressed and ITGB1 was knocked down simultaneously in native MDA-MB-231 cells treated with or without 12.5 ng/ml epirubicin **(G–O)**. **(G)** Cell proliferation analysis by CCK-8 assay. **(H)** Cell proliferation analysis by EdU assay. DAPI for nuclei. Scale bars: 100 μm. **(I)** Quantitative analysis of EdU assay. **(J)** Flow cytometry analysis of cell apoptosis. **(K)** Quantitative analysis of apoptotic cell percentages. **(L)** Cell migration analysis by wound healing assay. Scale bars: 200 μm. **(M)** Quantitative analysis of cell migration. **(N)** Cell invasion analysis by Transwell assay. Scale bars: 500 μm. **(O)** Quantitative analysis of cell invasion. OE-lnc005620+si-NC, overexpression of lnc005620 plus negative control; OE-lnc005620+si-ITGB1, overexpression of lnc005620 plus siRNA against ITGB1; OE-lnc005620+si-NC+Epi, overexpression of lnc005620 plus negative control and exposure to epirubicin; OE-lnc005620+si-ITGB1+Epi, overexpression of lnc005620 plus siRNA against ITGB1 and exposure to epirubicin. n = 3, Data are represented as the mean ± SD, *P < 0.05, **P < 0.01, ***P < 0.001.

To further investigate whether ITGB1 is a functional target of lnc005620, we first detected the alteration of ITGB1 expression after lnc005620 was overexpressed or knocked down in MDA-MB-231 cells. Immunofluorescence and western blotting experiments both showed that overexpression of lnc005620 upregulated the expression of ITGB1 (**Figures 6A–C**), whereas knockdown of lnc005620 downregulated the expression level (**Figures 6D–F**) whether the cells were treated with 12.5 ng/ml epirubicin for 48 h or not. Then, we modulated the expression of lnc005620 and ITGB1 simultaneously. By performing CCK-8 and EdU assays, we demonstrated that knockdown of ITGB1 alleviated lnc005620’s effect on cell proliferation and the results were more significant after treatment with 12.5 ng/ml epirubicin for 72 h (**Figures 6G–I**). Flow cytometry clearly showed that knockdown of ITGB1 partially abrogated the effects of lnc005620 on cell apoptosis and epirubicin treatment reinforced these effects (**Figures 6J, K**). Similarly, knockdown of ITGB1 also reversed the effect of lnc005620 on cell migration (**Figures 6L, M**) and invasion (**Figures 6N, O**) regardless of whether the cells were treated with epirubicin. In conclusion, lnc005620 may promote breast cancer progression and epirubicin resistance *via* ITBG1.

### lnc005620 Facilitates Tumorigenesis and Epirubicin Resistance *In Vivo*

**Figure 7:**
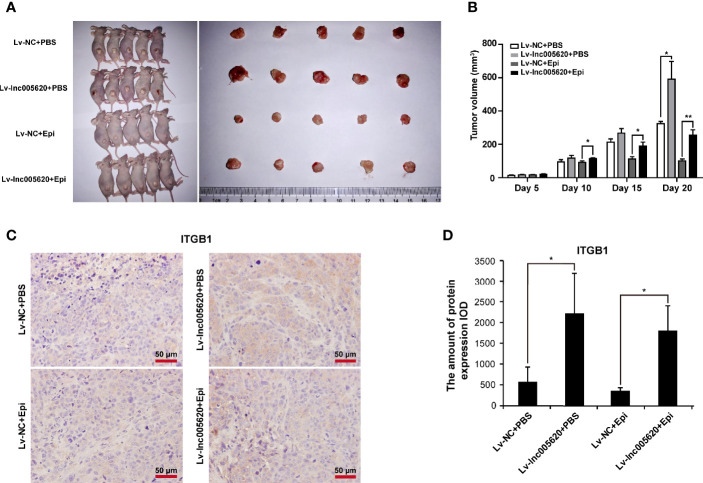
lnc005620 facilitates tumorigenesis and epirubicin resistance *in vivo*. MDA-MB-231 native cells stably transfected with Lv‐lnc005620 or negative control Lv‐NC were injected into mouse flanks. After the tumors were established, mice were treated with 5 mg/kg epirubicin or PBS subcutaneously at the tumor sites weekly for 2 weeks. **(A)** Images of nude mice and tumors. **(B)** Volumes of tumors. **(C)** Immunohistochemistry analysis of ITGB1 expression in the tumors. Scale bars: 50 μm. **(D)** Quantitative analysis of ITGB1 expression. n = 5/group. Data are represented as the mean ± SD, *P < 0.05, **P < 0.01.

To validate the *in vitro* results of lnc005620, we established a model of nude mice bearing MDA-MB-231 xenografts. MDA-MB-231 native cells stably transfected with Lv–lnc005620 or negative control Lv–NC were injected into the flanks of the mice. After the tumors were established, mice were treated with 5 mg/kg epirubicin or PBS subcutaneously at the tumor sites weekly for 2 weeks. Hence, four groups were established: Lv–NC+PBS, Lv–lnc005620+PBS, Lv–NC+epirubicin, and Lv–lnc005620+epirubicin. Tumors were removed, and the tumor mass was quantified (**Figure 7A**). The results showed that lnc005620 promoted tumor growth and that epirubicin treatment significantly suppressed tumor growth. More importantly, with treatment with epirubicin, tumor cells infected with Lv–lnc005620 grew faster than the controls, suggesting that lnc005620 suppressed the cell cytotoxicity induced by epirubicin treatment *in vivo* (**Figure 7B**). Moreover, immunohistochemistry analysis was conducted to determine whether lnc005620 affects the expression of ITGB1 in xenograft tumor tissues. As shown in **Figures 7C, D**, overexpression of lnc005620 promoted the level of ITGB1, indicating that lnc0065620 regulated carcinogenesis and epirubicin resistance by targeting ITGB1 in breast cancer.

## Discussion

Among various types of breast cancers, TNBC is frequently seen in young age (<50 years), at advanced stage at presentation, and at a higher rate of metastasis (37–39). Women with TNBC do not benefit from endocrine therapy or trastuzumab due to the lack of effective targets, ER, PR and HER2 (40). Chemotherapy is currently the mainstay of systemic medical treatment (41, 42). ANTs are commonly used chemotherapies for treating TNBC, especially metastatic TNBC, although they use should be carefully monitored in elderly cancer patients due to cardiotoxicity (43). Considering that the mean age at diagnosis of breast cancer in China is 45–55 years (44), ANTs are used more frequently in Chinese than in Western women. Disappointingly, the pathological complete response (pCR) rate is less than 30% due to the effect of chemotherapy resistance (41, 45). It is necessary to understand both molecular and cellular mechanisms and explore new targeted approaches in improving patient outcomes.

With the development of high-throughput technologies, various types of non-protein-coding transcripts in breast cancer have been studied (46). Although the question of whether noncoding RNAs represent ‘‘transcriptional noise’’ or truly functional biomolecules has been debated over the last decade (47), increasing studies have cemented lncRNAs as potent modulators and even diagnostic biomarkers of cancers (24). In our study, 12 differentially expressed lncRNAs and 42 TUCPs were identified between MDA-MB-231 cells resistant to epirubicin and their native controls using RNA sequencing. To identify novel lncRNAs involved in modulating the biological behavior of TNBC, a combination of bioinformatic analysis and experimental verification was used. In addition to being highly expressed in epirubicin-resistant cells and human TNBC tissues, aberrant expression of lnc005620 remarkably led to abnormal apoptosis, migration and invasion of MDA-MB-231 cells and partially abrogated the effects of epirubicin. Thus, lnc005620 was explored for the first time and validated to be a novel lncRNA that modulates oncogenesis and epirubicin resistance in TNBC.

The definition of lncRNAs based on length and function is widely accepted. However, the classifications of lncRNAs are currently confusing. One of the most commonly used and relatively convenient methods of classification relies on the corresponding genomic location and context of the lncRNA, that is, the position in the chromosome where the lncRNA is transcribed (21). lnc005620 is 2511 bp in length and an intronic lncRNA located on chromosome 7. Introns have long been known to harbor small ncRNAs such as snoRNAs, miRNAs and siRNA. ncRNAs within introns are commonly produced through the postsplicing process and are specific signals of gene transcription, impacting and modulating the expression of many other genes (48). Many of the long transcripts encoded within the introns of annotated genes have also been reported (49–51) and observed to be misregulated in cancers (52, 53). As an intronic lncRNA, the host gene of lnc005620 is DnajB6, a negative regulator of breast cancer (30). According to colocalization and coexpression gene analyses of lnc005620, we inferred the potential role of lnc005620 in breast cancer.

Since lnc005620 is a novel lncRNA, before we explored the underlying mechanisms, the subcellular location of lnc005620 was detected, which can help determine that the roles of lncRNAs depending on their mostly nuclear or cytoplasmic localization. The ultimate function of mRNAs is to be translated, so multiple layers of posttranscriptional regulation exist in the cytoplasm. lncRNAs can “identify” mRNAs in the cytoplasm and modulate their expression (54). In this study, we found that lnc005620 was mainly distributed in the cytoplasm of MDA-MB-231 cells. Clearly, proteomics, which is closely related to the phenotype, has a clear advantage over transcriptomics in investigating posttranslational modifications. Hence, we analyzed iTRAQ proteomics data and found 202 differentially expressed proteins between epirubicin-resistant MDA-MB-231 cells and the native controls. Three members of the integrin family, integrin β4, integrin β1, and integrin α6, were all upregulated in epirubicin-resistant cells. A high level of integrin β1 has been associated with poor outcomes and drug resistance in many types of tumors, including gastric cancer, pancreatic cancer, lung cancer, colon cancer and ovarian cancer (55–61). In triple-negative breast cancer, a high level of integrin β1 has also been considered a prognostic and predictive marker (34, 62). We confirmed that integrin β1 is regulated by lnc005620 and that lnc005620 promotes breast cancer progression and epirubicin resistance *via* integrin β1. Further research is needed to clarify the details of the interactions between lnc005620 and integrin β1 and also the other two members, integrin β4 and integrin α6.

In summary, our study revealed that novel lnc005620 promotes TNBC progression and chemoresistance to epirubicin by regulating integrin β1 expression both *in vitro* and *in vivo*. lnc005620 may be a promising therapeutic target for TNBC patients in terms of enhancing the benefits of epirubicin treatment.

## Data Availability Statement

The datasets presented in this study can be found in online repositories. The names of the repository/repositories and accession number(s) can be found below: https://www.ncbi.nlm.nih.gov/, GSE152003.

## Ethics Statement

The studies involving human participants were reviewed and approved by The Affiliated Obstetrics and Gynaecology Hospital of Nanjing Medical University. The patients/participants provided their written informed consent to participate in this study. The animal study was reviewed and approved by the Model Animal Research Center (Nanjing Medical University) Institutional Animal Care and Use Committee.

## Author Contributions

Data curation, FW, JT, and JY. Funding acquisition, FW. Investigation, SY, HY, and SG. Methodology, SY, FW, ML, and FC. Writing-original draft, FW. Writing-review and editing, JY and JT. All authors contributed to the article and approved the submitted version.

## Funding

This work was supported by grants from Jiangsu Provincial Medical Talent (QNRC2016102) to FW.

## Conflict of Interest

The authors declare that the research was conducted in the absence of any commercial or financial relationships that could be construed as a potential conflict of interest.
