# Supplementary material for: Novel Long Noncoding RNA 005620 Induces Epirubicin Resistance in Triple-Negative Breast Cancer by Regulating ITGB1 Expression
Source: Front Oncol. 2021 Mar 4;11:592215. doi: 10.3389/fonc.2021.592215 (PMC7970185; doi:10.3389/fonc.2021.592215)
Supplement: Supplementary file 1 [file DataSheet_1.zip › Data Sheet 1/Supplementary_Data_final.docx]

**Supplementary Data**

**Supplementary Methods**

**RNA isolation, library preparation, sequencing and data analysis**

A total amount of 20 ng RNA per sample was used as input material for the RNA sample preparations. First, ribosomal RNA was removed by an Epicentre Ribo-zero™ rRNA Removal Kit (Epicenter, WI, USA), and the rRNA-free sample was cleaned up by ethanol precipitation. Subsequently, sequencing libraries were generated using the rRNA-depleted RNA with the NEBNext® Ultra™ Directional RNA Library Prep Kit (NEB, Ipswich, UK) following the manufacturer’s recommendations. The libraries were then sequenced on an Illumina HiSeq^TM^ 2000 platform (Illumina, CA, USA), and 125 bp paired-end reads were generated.

Raw data (raw reads) of FASTQ format were first processed through in-house Perl scripts, and clean data (clean reads) were obtained. Paired-end clean reads were aligned to the human genome (hg19) using TopHat version 2.012. The mapped reads of each sample were assembled by Cufflinks software, version 2.2.1 into transcripts. Cuffmerge was used to merge all of the identified transcripts into an integrated transcriptome. Cuffquant was used to calculate the fragments per kilobase per million mapped reads (FPKMs) of both lncRNAs and coding transcripts in each sample. We filtered out the transcripts expressed at low levels and retained transcripts with FPKM < 0.01 in >75% of the samples.

Novel lncRNAs among noncoding transcripts were identified according to the following method. Only transcripts with a minimum length of 200 nt were retained. Transcripts located 2000 bp away from any known gene were selected based on RefSeq genes, Ensembl genes, and GENCODE genes. CNCI (Coding-Non-Coding-Index) (v2), Pfam Scan (v1.3) and PhyloCSF (phylogenetic codon substitution frequency) (v20121028) were all used for coding potential analysis. Transcripts predicted to have coding potential by any/all of the four tools above were filtered out, and those without coding potential were our candidate set of lncRNAs.

The Cuffdiff tool in the Cufflinks software package provided statistical routines for determining differential expression and was used to identify differentially expressed transcripts between epirubicin-resistant and native MDA-MB-231 cells. Transcripts with a *P*-adjust < 0.05 or *P* value < 0.05 were considered differentially expressed.

To analyze coexpressed genes of differentially expressed transcripts, both trans (pairs consisting of transcripts located >1 Mb away from each other or located on different chromosomes) and cis (pairs consisting of transcripts located within a genomic window of 100 kb) correlations were calculated for Pearson’s correlation coefficients using FPKM expression estimates. We defined coding and noncoding transcripts as linked only when their associated Pearson’s correlation coefficient was significantly greater than 0.9 (*P* < 10^-5^).

**Protein extraction, iTRAQ proteome and data analysis**

*Protein extraction* - lysis buffer (7 M urea and 40 mM Tris-HCl containing 1 mM PMSF, 2 mM EDTA, and 10 mM DTT, pH 8.5) was added to the samples, which were then ultrasonicated for 5 min. After centrifugation at 13000 ×g for 20 min at 4°C, four volumes of 10% TCA/acetone were added to the supernatant to precipitate proteins at -20°C overnight. The precipitated proteins were air-dried, resuspended in lysis buffer, and centrifuged. The resulting supernatants were incubated at 56°C with 10 mM DTT for 30 min to reduce the proteins. The protein concentration was determined by the Bradford method using bovine serum albumin (BSA) as the standard.

*Trypsin digestion and iTRAQ labeling* - protein solutions (100 μg) were diluted 4-fold with 100 mM TEAB (tetraethylammonium bromide), after which proteins were digested overnight with Trypsin Gold (Promega, WI, USA) at 37°C (40:1, protein: trypsin). The peptides were desalted and then vacuum-dried according to the protocol recommended by the manufacturer. Peptide samples were labeled using iTRAQ 8-plex kits (AB SCIEX, MA, USA).

*High-pH reversed-phase chromatography* - an Ultimate3000 HPLC system (Thermo Fisher) equipped with 4.6-mm-inner diameter × 250-mm-long Durashell C18 columns (5 μm, 100 Å, Agela, Tianjin, China) was used for high-pH fractionation. In total, 12 fractions were pooled and dried by vacuum centrifugation for subsequent nano-reversed phase liquid chromatography (nano-LC) fractionation.

*RPLC-MS/MS analysis* - each fraction was suspended in loading buffer (0.1% FA, 2% ACN) and separated using an Eksigent nano-LC system (AB SCIEX) equipped with a C18 reversed-phase column (75-μm inner diameter, 15-cm long, 3-μm resin, Agela). Then, the eluent was transferred to TripleTOF™ 5600+ (AB SCIEX) containing a multichannel TDC detector with four-anode channel detection.

*Proteomic data analysis* - for protein identification, a mass tolerance of 0.05 Da (50 ppm) was permitted for intact peptide masses and 0.1 Da for fragmented ions, with allowance for one missed cleavage in the trypsin digests. The peptide data were analyzed using ProteinPilot^TM^ V4.5 software (AB SCIEX). Data with a false discovery rate (FDR) of less than 1% were used for the ProtScore (unused) measure. Peptides with scores of over 1.3 (confidence over 95%) were chosen. For quantitative changes, a 1.5-fold cutoff was set to determine upregulation and downregulation.

**Fluorescence in situ hybridization (FISH)**

Cells were fixed in 4% formaldehyde for 10 minutes and then washed with PBS. The fixed cells were treated with pepsin and dehydrated with ethanol. Cells were then incubated with 20 µM Ribo^TM^ lncRNA FISH Probe Mix (Ribo) in hybridization buffer overnight. After hybridization, the slides were washed, dehydrated and mounted with DAPI (C1002, Beyotime) for detection. The slides were visualized for immunofluorescence with an UltraVIEW® VoX system (PerkinElmer, IL, USA). Small nuclear RNA U6 (U6) and 18S ribosomal RNA (18S) served as references for nuclear and cytoplasmic localization, respectively.

**GO and KEGG analyses**

Gene Ontology (GO) and KEGG analyses were used to categorize the differentially expressed genes or lncRNA target genes into families and subfamilies according to their functions and to identify pathways that are enriched in these genes. GO analysis was implemented by the GOseq R package, in which gene length bias was corrected. GO terms with corrected *P* values less than 0.05 were considered significantly enriched in differentially expressed genes. KOBAS software v3.0 was used to test the statistical enrichment of differentially expressed genes or lncRNA target genes in KEGG pathways.

**Immunohistochemistry and immunofluorescence**

For immunocytochemistry staining, sections were incubated with anti-integrin β1 antibody (1:200, 26918-1-AP, 12594-1-AP, Proteintech, IL, USA) for 30 min at room temperature. The signals were detected by using horseradish peroxidase-conjugated IgG (goat anti-rabbit, 1:200, AS1107, Aspen Biotechnology, Wuhan, China) in combination with the ABC kit (Vector Laboratories, CA, USA) and DAB Substrate (Vector Laboratories). Samples were visualized and imaged under microscopy (Olympus cx41). ImageJ V1.8.0 was used for quantitative analysis.

For immunofluorescence, cells were fixed in 4% formaldehyde for 15 minutes and then washed with PBS. The fixed cells were permeabilized in 0.2% Triton X‐100 (Beyotime) for 10 minutes and washed with PBS again. Then, 10% goat serum was used for blocking, and the cells were incubated with anti-integrin β1 antibody (1:200, 26918-1-AP, Proteintech) overnight at 4°C and then incubated with goat anti-rabbit secondary antibody (1:200, Alexa Fluor® 488, Ab150077, Abcam, MA, USA) for 1 hour. The cells were then washed and incubated with DAPI (Beyotime) for nuclear staining and immunofluorescence was visualized with an UltraVIEW® VoX system (PerkinElmer).

**Reverse transcription‐quantitative polymerase chain reaction (RT‐qPCR)**

The PCR conditions were as follows: 10 min at 95°C, followed by 40 cycles of 15 s at 95°C and 60 s at 60°C. The temperature was increased from 60°C to 95°C, and the PCR melting curve was generated every 1.0°C after the amplification reaction (MyCycle, Bio-Rad, CA, USA). The mean value of triplicates for each sample was calculated and expressed as the cycle threshold (Ct). Gene expression was then calculated as the ΔCt, the difference between the Ct value of the sample and the Ct value of GAPDH, which was used as an internal reference. The relative expression level was evaluated using the comparative delta-delta Ct method (2^-ΔΔCt^). PCR primers were designed by Primer 5 (PREMIER Biosoft, CA, USA) and synthesized by GeneCreat. The sequences are shown as follows:

lnc005620 (Fw) 5’-TGAAGACATCATCTGGGTTACG-3’, (Rv) 3’-CAGTGCTAAG

AAACGGGACAT-5’; GAPDH (Fw) 5’-ACAACTTTGGTATCGTGGAAGG-3’, (Rv) 3’-GCCATCACGCCACAGTTTC-5’.

**Protein extraction and western blotting**

Aliquots (60 μg) of proteins were separated by 12% sodium dodecyl sulfate (SDS)-polyacrylamide gel electrophoresis and transferred onto a poly (vinylidene difluoride) membrane (Merck Millipore, MA, USA). The membrane was blocked with 1% (w/v) BSA (Thermo Fisher Scientific, MA, USA) in TBST (10 mM Tris-HCl, pH 7.8, 150 mM NaCl, and 0.1% TWEEN 20) for 2 h and then incubated with primary polyclonal antibody (anti-integrin β1, 26918-1-AP, 1:500 or anti-GAPDH, 60004-1-Ig, 1:20000) (Proteintech) in TBST containing 1% (w/v) BSA overnight at 4°C. The blots were treated with horseradish peroxidase-conjugated IgG (goat anti-rabbit, 1:200, AS1107; goat anti-mouse, 1:200, AS1106, Aspen Biotechnology) in TBST containing 1% (w/v) BSA for 60 min, and the immune complexes were detected by using an ECL plus detection kit (Cell Signaling Technology, MA, USA). Bands were quantified by using densitometric image analysis software (Quantity One; Bio-Rad, CA, USA).

**Supplementary Tables**

Supplementary Table 1. Full length of lnc005620

| **lnc005620 2511 bp (5’-3’)** |
| --- |
| CGCATTTCGGAAGCAAGGCTTCTGCCTGCAAAACACCACTTTCATCCTTTGTAATCGTAATGTTGAAGAATTGCTTTTATTTCTAGTCAGTCTTAACGGTGCAACTTACTGGCGCTTACAAAGGACAGCAGGCGAGCTGTATTCAATATGCTTATGTTTTATTTATGTAGGTGGCATTTAAAATACATGATGTGTTTAGGGTTACATTGTCCACAGAAAGCATCAAATACCACTCCTCTCCCCGCCAAAACCAAATAAACAAAGCCAACTCTTTGGCAACAGTTGTGTTAAATAAAATCCCAGGTCACACTTGTTTCTGGCTCCCAAGCCTGGGTCACTGCTACATGGATTGCGCCAAAAAATTCCCAGCTTCAACACTGCTAGATTAAAATTGCTGGCATTTTTAAATCACAGCAAAGCTTTTCACAATGCCCTCAAGTCCAAGAGGACAAAGGAGAAAGCAACATGAACGGCAGATCCTCATGTGAAAGGGAAGGAAAGTCACTGGGAGGGAGCATGCAGGGAAGAAGTCAAGGCAGCCCTGGAATTCTACTCCGTGCTCAATAAAAACAAAACGTGAAGAAGCAATACATCATGCAAACGAAATAATGACCGGAAGTGGGCGCATCTAGTTAGAATGAAGTGACTTTCGTAAGGAGTCAATGTTCGCGAACTGAAACATGAGTTCAACCTCCTTGTGCCGTCTCTGGGTGTTTTGCGCGTGTGTAAATACCGGCCCGTTTTCCCCAGCATGGCCCTAACCCATGGACACTAGGGAGAGTGCCACTGAGCAAAAATTTTTTGCTAAAAATAATTAGCAAAAATCCAAGAAAAAAATATGGAATCTAGCAAAACCTACACCATATTTGAAGTTGAATTTGCACTACCTGCAAAGGATGAAAATTAAAAGAGTAAACAGAAGGAATGATTTTACATCTGATGTGATTCACGACAGCCTTCTATTCGTGTGGCCGGATTGCTCTGAGGAAAAGGTGCCCTGACGGGCAGGATTCAATGTGTCACTTGCAATATTCTCTAAGTTACACTCTACTGAATTACCTTCTAATTCCAAACACTACGCCGCCACAGAAAGCCTCATTTCATGAATGTTACTCAGTTAGCCATCTGCGCCCTCCTGTACTTCTAAAGCCAGAAATATTCCAGCAGCATGCGGAGTGGTTGAGTAGCCGAAAAGAATGAAGAGGATCCGGAAGCACTAGAGGAAAATGCAGCAGATTGAGAGCTGGAGAGAAGCAGAGAGTCGCGGCCAAGTCCCAAACGTCTGTCCCCGCCACCCGTGCCGTCAGCCCCGTCTCCTGACCCTGCTCTGACTGATCCGTGATCGCATAATCCTGCCGAGCGAGTGTGGCCTCGAGACAGTCCTGACCGACGCTGCTACCTAGCGCGTGTGCACGACCGCATGCCGAGCACGGTGGAGCGCCAGCGTCTGGGGGTGCACCGCGTGCCTCAAGTCCGGTCTAGTGATTGCCTTTGGTCGACTTCTTCTTCTTCGACTCCTCTCTCTGCTTCTGCTTCTTCCTCTTGCCACCTTCTTTCAATCCTGCTGCGGACGCGAGGGGGTCCCAGGGCCCGGGTGCCCGAGGTCGGTCCTGCTCGCCCTCCTCCTCAGAGAGACAGTGAGGCGCGTGTCTCAGCAGCGAGGCAGGCCGGGGCGGCTTCGGCGGGCGGAGGCCGGCAGGCTGGGCTGGCAGGGCGTTCTGGCCTCTCCGCATGCGCTCCTCAGCGAGGGCATCGTCGTCGGCCACACCATTTATTGTTAAGGACTTTAACTGGCCATCTTCTTCAACTTCTACTCTTTCTTGACCGTTCTCGACAATTCTCTTTGTAGTGATTTTTCTGCCATTAACCATTTTAGTTGAAGTTGATATCGATTTGAAGTTGCCCATGCCACTACCACCAAATGACGTGGAAGAGAATGAAGTGAGGCCCCCGTGACCTAGTGACCCAAATGAAGTAAATCCTGTATCAAAAGAAGAAAATCCACTTCCAAAAGACGGAAATCCACTGAACGCAGAGAAAAACGACCCCGTCCCTCGGCTTCTGCTTCCTCGGGGACCCCTTCGATTCCCAAAGAAGTCCTCAAAAGGGTCTTCAAAGAAGTCAAATGAAAATGGGTCCCTTCCACCAAAAAATTCCCTGAAGACATCATCTGGGTTACGGAATGTGAAGCCAAATTCAAATGGACTGTCAAAATGACTTCCACCTCCTCCTCCACCATTTAATCCTTCTTTGCCATATTTGTCATAGATGTCCCGTTTCTTAGCATCCGACAGCACTTCATATGCCTCCGCTACTTGCTTGAATTTTCTCTCTGCTTCTTCTTTATTCTCAGGATTTTTATCTGGATGCCACTTCAGTGCCAGTTTCCGATATGCCTTTTTAATATCCTCGGGTGAGGCATGTCTCTGCACGCCTAGAACTTCATAGTAATCCACCATGTTTTACGAGATTGTTGGAATGGGTCCTGCTTCTTCTCCCTCCGAACTTGGC |

Supplementary Table 2. siRNA sequences targeting lnc005620 and ITGB1

| **Name** | **Sequence (5’-3’)** |
| --- | --- |
| si-LNC_005620-1-s | CCACUUUCAUCCUUUGUAATT |
| si-LNC_005620-1-a | UUACAAAGGAUGAAAGUGGTT |
| si-LNC_005620-2-s | GCAGGCGAGCUGUAUUCAATT |
| si-LNC_005620-2-a | UUGAAUACAGCUCGCCUGCTT |
| si-LNC_005620-3-s | CCGGUCUAGUGAUUGCCUUTT |
| si-LNC_005620-3-a | AAGGCAAUCACUAGACCGGTT |
| si-LNC_005620-4-s | GCUUCUGCUUCUUCCUCUUTT |
| si-LNC_005620-4-a | AAGAGGAAGAAGCAGAAGCTT |
| si-ITGB-s | AGAGUGCCGUAACAACUGUGGTT |
| si-ITGB-a | CCACAGUUGUUACGGCACUCUTT |
| NC-s | UUCUCCGAACGUGUCACGUTT |
| NC-a | ACGUGACACGUUCGGAGAATT |

siRNA: small interfering RNA; NC: negative control

Supplementary Table 3. Sample comparison to the DSMZ STR profile database

| **Marker** | **Sample** | | | **DSMZ** | | | |
| --- | --- | --- | --- | --- | --- | --- | --- |
|  | **Allele1** | **Allele2** | **Allele3** | | **Allele1** | **Allele2** | **Allele3** |
| D5S818 | 13 | 13 |  | | 12 | 12 |  |
| D13S317 | 13 | 13 |  | | 13 | 13 |  |
| D7S820 | 8 | 9 |  | | 8 | 9 |  |
| D16S539 | 12 | 12 |  | | 12 | 12 |  |
| VWA | 15 | 18 |  | | 15 | 18 |  |
| TH01 | 7 | 10 |  | | 7 | 9.3 |  |
| AMEL | X | X |  | | X | X |  |
| TPOX | 8 | 9 |  | | 8 | 9 |  |
| CSF1PO | 12 | 13 |  | | 12 | 13 |  |
| D21S11 | 33.2 | 33.2 |  | |  |  |  |

Supplementary Table 5. Clinical characteristics of 22 participants

| **No.** | **Age** | **Meno-**  **pause** | **Path-**  **ology** | **Tumor size (cm)** | **Lymph nodes** | **TNM** | **ER** | **PR** | **Her2** | **Ki67 (%)** |
| --- | --- | --- | --- | --- | --- | --- | --- | --- | --- | --- |
| 1 | 54 | Y | IDC | 1.5 | 0/1 | I | - | - | - | 80 |
| 2 | 47 | N | IDC | 2.5 | 0/15 | IIA | - | - | - | 40 |
| 3 | 37 | N | MC | 1.9 | 2/23 | IIA | - | - | - | 80 |
| 4 | 49 | Y | IDC | 1.8 | 1/25 | IIA | - | - | - | 70 |
| 5 | 48 | N | IDC | 2.5 | 0/35 | IIA | - | - | - | 80 |
| 6 | 34 | N | IDC | 1.5 | 0/2 | I | - | - | - | 60 |
| 7 | 56 | Y | IDC | 1.9 | 0/5 | I | - | - | - | 15 |
| 8 | 65 | Y | IDC | 1.6 | 0/3 | I | - | - | - | 30 |
| 9 | 57 | Y | IDC | 2.9 | 0/4 | IIA | - | - | - | 10 |
| 10 | 60 | Y | IDC | 2.2 | 0/6 | IIA | - | - | - | 10 |
| 11 | 46 | N | IDC | 1.6 | 0/3 | I | ++ | + | +++ | 70 |
| 12 | 41 | N | IDC | 1 | 1/33 | IIA | +++ | + | + | 50 |
| 13 | 63 | Y | IDC | 1.5 | 0/3 | I | +++ | + | +++ | 30 |
| 14 | 59 | Y | IDC | 1.3 | 0/4 | I | +++ | +++ | - | 25 |
| 15 | 48 | N | IDC | 2.1 | 0/3 | IIA | +++ | +++ | + | 15 |
| 16 | 58 | Y | IDC | 2.0 | 1/12 | IIA | +++ | ++ | - | 10 |
| 17 | 36 | N | IDC | 1.4 | 1/2 | IIA | +++ | +++ | + | 65 |
| 18 | 42 | N | IDC | 1.5 | 3/22 | IIA | +++ | +++ | - | 70 |
| 19 | 47 | N | IDC | 1.0 | 0/6 | I | +++ | ++ | + | 5 |
| 20 | 30 | N | IDC | 1.4 | 0/4 | I | - | - | +++ | 40 |
| 21 | 51 | N | IDC | 1.8 | 1/4 | IIA | ++ | ++ | - | 10 |
| 22 | 43 | N | IDC | 1.9 | 0/18 | I | +++ | +++ | - | 15 |

IDC: invasive ductal carcinoma; MC: medullary carcinoma; ER: estrogen receptor; PR: progesterone receptor; Her2: human epidermal growth factor receptor-2; Y: yes; N: no

Supplementary Table 7. Clinical characteristics of 12 participants

| **Type** | **Age** | **Meno-**  **pause** | **Path-**  **ology** | **Tumor size (cm)** | **Lymph nodes** | **TNM** | **ER** | **PR** | **Her2** | **Ki67 (%)** | **metastasis** |
| --- | --- | --- | --- | --- | --- | --- | --- | --- | --- | --- | --- |
| TNBC  nonresponse | 60 | Y | IDC | 1.6 | 0/27 | I | - | - | - | 30 | lung |
|  | 43 | N | IDC | 4.0 | 27/27 | IIIC | - | - | - | 15 | liver |
|  | 54 | Y | IDC | 2.5 | 26/33 | IIIC | - | - | - | 30 | brain |
| TNBC  response | 46 | N | IDC | 4.0 | 0/9 | IIA | - | - | - | 3 | / |
|  | 58 | Y | IDC | 1.2 | 0/17 | I | - | - | - | 60 | / |
|  | 43 | N | IDC | 4.0 | 0/29 | IIA | - | - | - | 80 | / |
| non-TNBC  nonresponse | 62 | Y | IDC | 3.0 | 13/22 | IIIC | +++ | - | - | 60 | Lung/  mediastinum |
|  | 46 | N | IDC | 2.0 | 2/16 | IIA | ++ | ++ | + | 30 | SCLN |
|  | 44 | N | IDC | 2.0 | 0/20 | IIA | - | - | + | 40 | lung |
| non-TNBC  response | 38 | N | IDC | 2.0 | 0/23 | I | + | + | - | 20 | / |
|  | 46 | N | IDC | 1.5 | 1/28 | IIA | + | ++ | - | 10 | / |
|  | 50 | N | IDC | 1.5 | 0/22 | I | +++ | ++ | + | 10 | / |

TNBC: triple-negative breast cancer; IDC: invasive ductal carcinoma; ER: estrogen receptor; PR: progesterone receptor; Her2: human epidermal growth factor receptor-2; SCLN: supraclavicular lymph node; Y: yes; N: no

**Supplementary Figures**


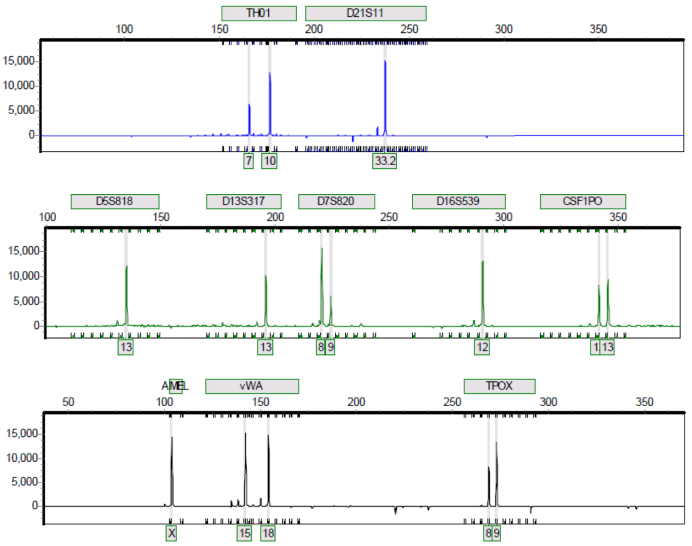


**Supplementary Figure 1.** STR profiles of the cell samples.


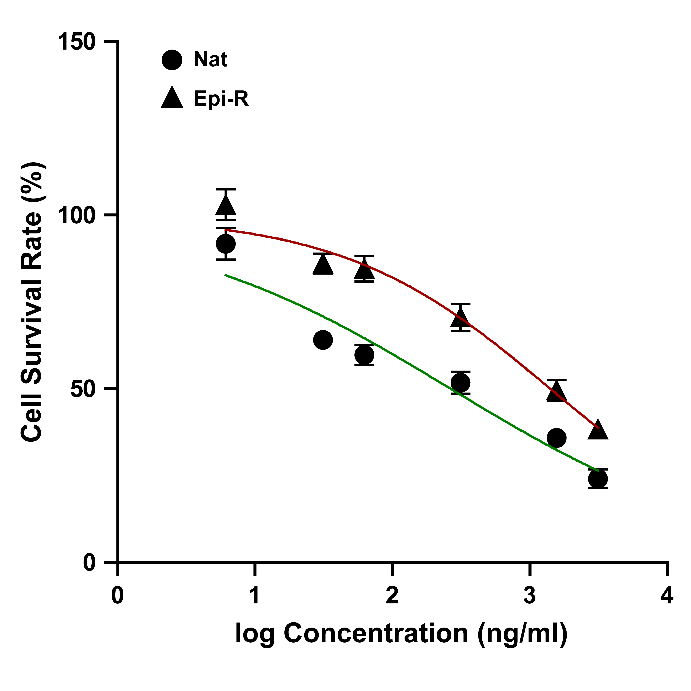


**Supplementary Figure 2.** **Cell viability of epirubicin-resistant (Epi-R) and native (Nat) MDA-MB-231 cells.** Epi-R and Nat cells were exposed to epirubicin at concentrations of 6.25 ng/ml, 31.25 ng/ml, 62.5 ng/ml, 312.5 ng/ml, 625 ng/ml, and 3125 ng/ml for 48 h. The cell survival rate detected by MTT assay decreased with increasing concentrations of epirubicin. n = 3. Data are represented as the mean ± SD.


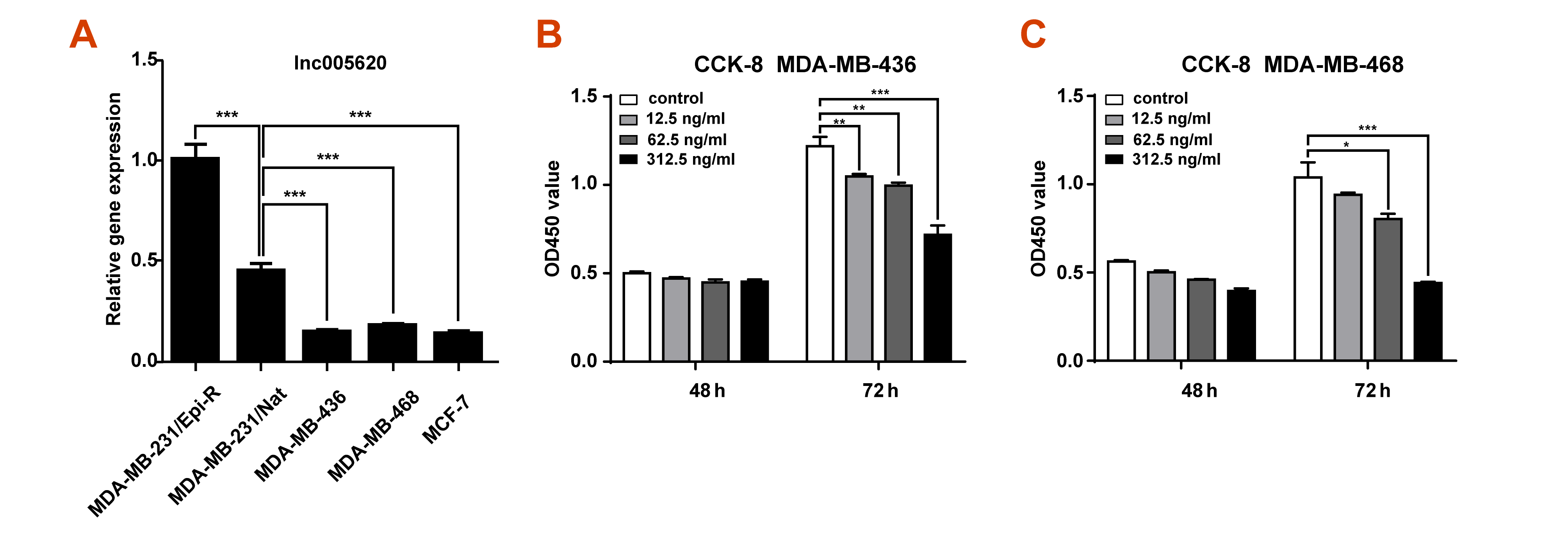


**Supplementary Figure 3. Cell viability of MDA-MB-436 and MDA-MB-468 cells after exposure of doxorubicin (Dox).** MDA-MB-436 and MDA-MB-468 cells were exposed to different concentrations of doxorubicin (12.5 ng/ml, 62.5 ng/ml and 312.5 ng/ml). **A.** RT-qPCR validation of lnc005620 expression in Epi-R and Nat MDA-MB-231 cells, MDA-MB-436, MDA-MB-468 and MCF-7 cells. **B.** Cell proliferation analysis by CCK-8 assay in MDA-MB-436 cells. **C.** Cell proliferation analysis by CCK-8 assay in MDA-MB-468 cells. n = 3. Data are represented as the mean ± SD, **P* < 0.05, ***P* < 0.01, ****P* < 0.001.


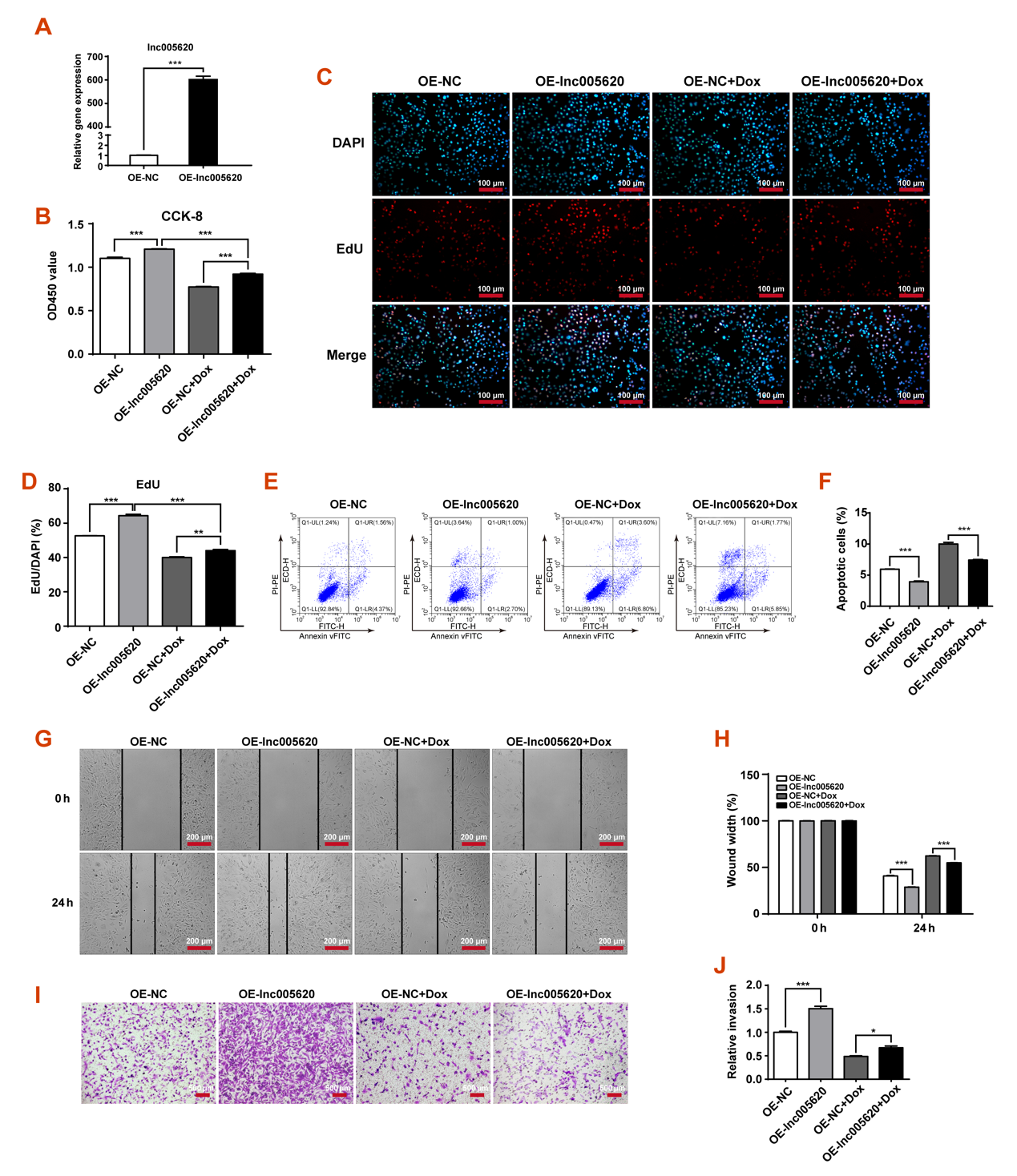


**Supplementary Figure 4. lnc005620 promotes proliferation, invasion and doxorubicin resistance in MDA-MB-436 cells.** lnc005620 was overexpressed in native MDA-MB-436 cells treated with or without 62.5 ng/ml doxorubicin. **A.** RT-qPCR validation of lnc005620 expression. **B.** Cell proliferation analysis by CCK-8 assay. **C.** Cell proliferation analysis by EdU assay. DAPI for nuclei. Scale bars: 100 μm. **D.** Quantitative analysis of EdU assay. **E.** Flow cytometry analysis of cell apoptosis. **F.** Quantitative analysis of apoptotic cell percentages. **G.** Cell migration analysis by wound healing assay. Scale bars: 200 μm. **H.** Quantitative analysis of cell migration. **I.** Cell invasion analysis by Transwell assay. Scale bars: 500 μm. **J.** Quantitative analysis of cell invasion. OE-NC, negative control; OE-lnc005620, overexpression of lnc005620; OE-NC+Dox, negative control and exposure to doxorubicin; OE-lnc005620+Dox, overexpression of lnc005620 and exposure to doxorubicin. n = 3. Data are represented as the mean ± SD, **P* < 0.05, ***P* < 0.01, ****P* < 0.001.


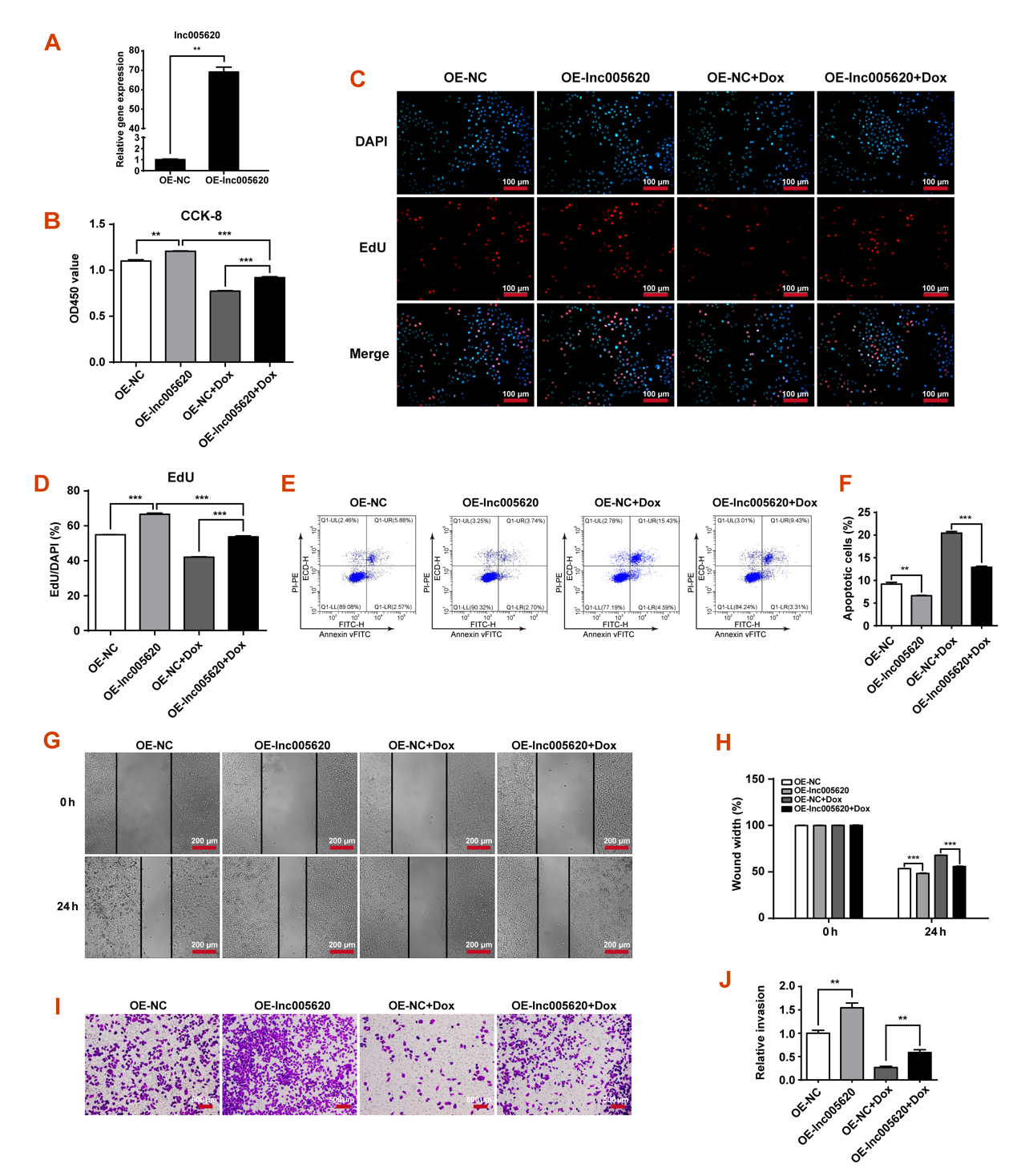


**Supplementary Figure 5. lnc005620 promotes proliferation, invasion and doxorubicin resistance in MDA-MB-468 cells.** lnc005620 was overexpressed in native MDA-MB-468 cells treated with or without 62.5 ng/ml doxorubicin. **A.** RT-qPCR validation of lnc005620 expression. **B.** Cell proliferation analysis by CCK-8 assay. **C.** Cell proliferation analysis by EdU assay. DAPI for nuclei. Scale bars: 100 μm. **D.** Quantitative analysis of EdU assay. **E.** Flow cytometry analysis of cell apoptosis. **F.** Quantitative analysis of apoptotic cell percentages. **G.** Cell migration analysis by wound healing assay. Scale bars: 200 μm. **H.** Quantitative analysis of cell migration. **I.** Cell invasion analysis by Transwell assay. Scale bars: 500 μm. **J.** Quantitative analysis of cell invasion. OE-NC, negative control; OE-lnc005620, overexpression of lnc005620; OE-NC+Dox, negative control and exposure to doxorubicin; OE-lnc005620+Dox, overexpression of lnc005620 and exposure to doxorubicin. n = 3. Data are represented as the mean ± SD, ***P* < 0.01, ****P* < 0.001.


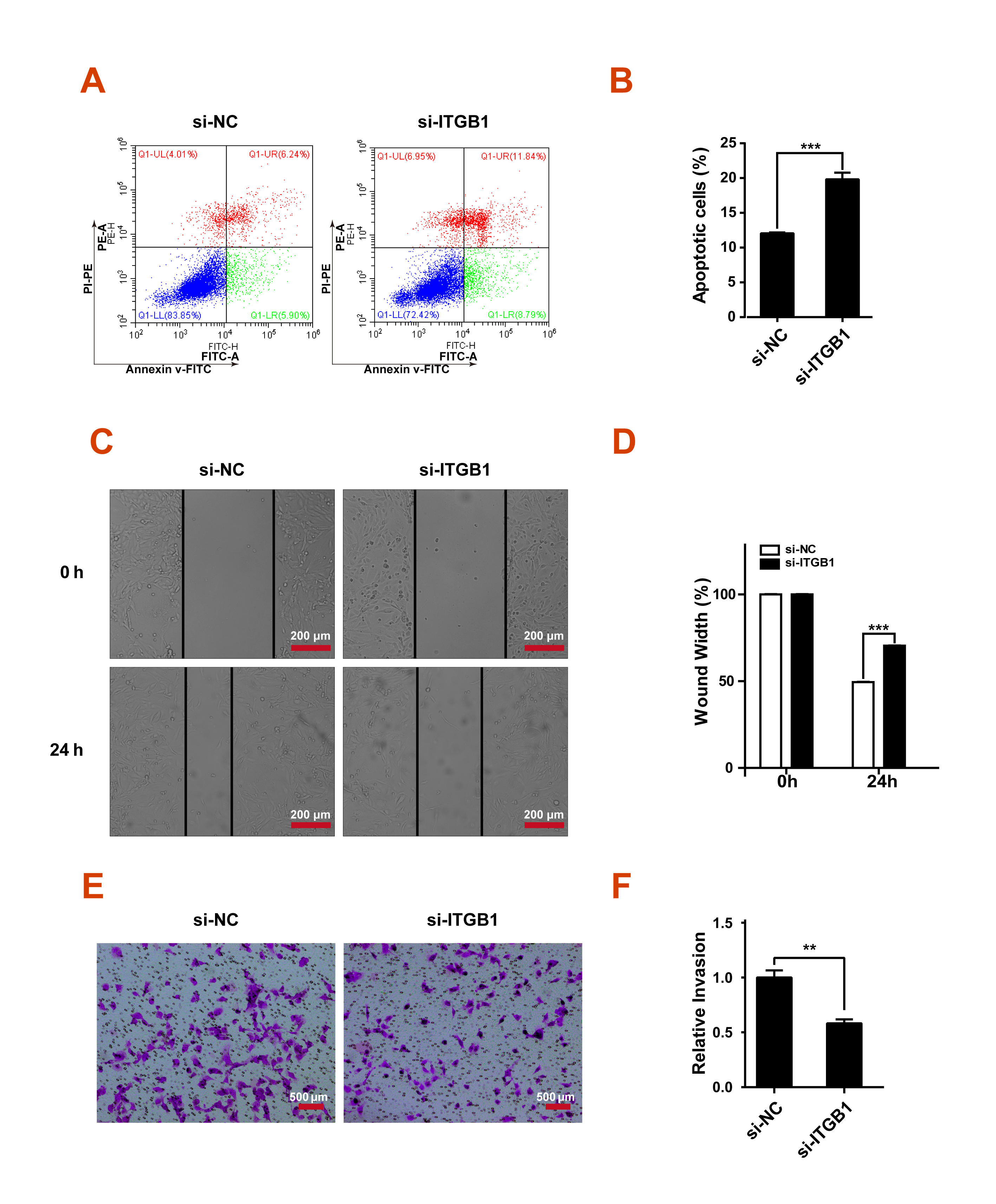


**Supplementary Figure 6.** **Inhibition of ITGB1 promotes apoptosis and inhibits the migration and invasion of epirubicin-resistant MDA-MB-231 cells. A.** Flow cytometry analysis of cell apoptosis. **B.** Quantitative analysis of apoptotic cell percentages. **C.** Cell migration analysis by wound healing assay. Scale bars: 200 μm. **D.** Quantitative analysis of cell migration. **E.** Cell invasion analysis by Transwell assay. Scale bars: 500 μm. **F.** Quantitative analysis of cell invasion. si-NC, negative control; si-ITGB1, siRNA targeting integrin β1. n = 3. Data are represented as the mean ± SD, ***P* < 0.01, ****P* < 0.001.
